# Puerarin Delays the Progression of Muscle Atrophy in Mice With Dexamethasone‐Induced Sarcopenia Through Inhibiting the TNF‐α/NF‐κB Pathway

**DOI:** 10.1002/fsn3.70166

**Published:** 2025-04-18

**Authors:** Shangjin Lin, Ying Cheng, Xiuxiu Chen, Fengjian Yang, Yongqian Fan, Shengwu Yang

**Affiliations:** ^1^ Department of Orthopedics The First Affiliated Hospital of Wenzhou Medical University Wenzhou China; ^2^ Shanghai Key Laboratory of Clinical Geriatric Medicine Shanghai China; ^3^ Department of Gastroenterology Huadong Hospital Affiliated to Fudan University Shanghai China; ^4^ Department of Orthopedics Huadong Hospital Affiliated to Fudan University Shanghai China

**Keywords:** muscle atrophy, NF‐κB, Puerarin, sarcopenia, TNF‐α

## Abstract

Sarcopenia, marked by the loss of muscle mass and function, is a chronic condition that worsens with age. Currently, there are no effective drugs for its treatment. Puerarin, a potent natural compound extracted from the root of *Pueraria lobata* , exhibits various pharmacological properties, including anti‐inflammatory, antioxidative, and anti‐apoptotic effects. It remains unclear whether puerarin possesses anti‐muscle atrophy capabilities. This study aims to evaluate the effectiveness of puerarin in delaying the development of muscle atrophy in mice with dexamethasone‐induced sarcopenia and to explore the underlying molecular mechanisms. Experimental findings reveal that puerarin effectively alleviates a range of physiological and behavioral changes caused by dexamethasone, including weight loss, deterioration in muscle mass and function, and destruction of the ultrastructure of muscle fibers. Notably, puerarin significantly enhances muscle mass and function in mice with dexamethasone‐induced sarcopenia, reduces the release of pro‐inflammatory cytokines while promoting the production of anti‐inflammatory factors, lowers oxidative stress, inhibits the expression of muscle apoptosis proteins, and decelerates muscle atrophy development by suppressing the TNF‐α/NF‐κB signaling pathway. In conclusion, these findings not only further confirm the potential value of puerarin as a therapeutic drug for sarcopenia but also provide new directions and theoretical foundations for future research.

## Graphical abstract

Puerarin alleviates dexamethasone‐induced muscle atrophy in mice by inhibiting the TNF‐α/NF‐κB signaling pathway. This suppression downregulates the expression of Atrogin‐1 and MuRF‐1, key mediators of protein degradation. The findings suggest that puerarin may serve as a potential therapeutic agent for sarcopenia.

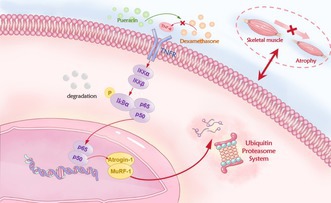

## 1 Introduction

With the escalating severity of population aging, age‐associated chronic conditions are emerging as critical areas necessitating urgent public health focus. Notably, sarcopenia stands out as a significant concern within this context. Sarcopenia, also known as muscle wasting syndrome, is a progressive, systemic skeletal muscle disease characterized by the accelerated loss of skeletal muscle mass and function (Dao et al. 2020). Although sarcopenia is not a direct cause of mortality, it significantly impacts the quality of life in the elderly. The loss of skeletal muscle mass and function is an inevitable event in the normal aging process, increasing the risk of adverse outcomes such as falls, fractures, physical disability, and even death among older adults (Argiles et al. 2015).

The mechanisms underlying the onset and progression of sarcopenia are complex and not fully elucidated, involving numerous interacting molecular biological processes. Current research indicates that these processes include neuromuscular junction degeneration, declines in sex and growth hormone levels, insulin resistance, imbalances in protein metabolism, mitochondrial dysfunction, chronic inflammation, and increased oxidative stress (Marty et al. 2017; Riuzzi et al. 2018; Alizadeh et al. 2022; Zhao et al. 2023; Barbiera et al. 2020; Hashemi et al. 2016). The persistent increase in chronic inflammation and oxidative stress has a close and complex relationship with sarcopenia, playing a key role in its pathogenic mechanisms.

Inflammatory cytokines play a pivotal role in the aging of skeletal muscle and the development of sarcopenia. Specifically, interleukin‐6 (IL‐6) and tumor necrosis factor‐alpha (TNF‐α) have been demonstrated to significantly increase the rate of skeletal muscle cell aging and occupy a critical position in the complex network that links inflammatory signals with aging (Foreman et al. 2021). Crucially, the progression of chronic inflammation and aging is not solely dependent on the increase in pro‐inflammatory cytokine concentrations but is also influenced by the decline in anti‐inflammatory cytokine levels (Thoma and Lightfoot 2018). This dysregulation of inflammatory signals not only promotes direct damage to skeletal muscle cells but also activates various signaling pathways, accelerating the loss of muscle mass and decline in strength. Furthermore, oxidative stress is considered one of the key factors promoting the loss of skeletal muscle mass and decline in function (Li et al. 2003). Studies have found that reactive oxygen species (ROS) can promote the ubiquitination of muscle proteins by increasing the expression of E3 ligases (Atrogin‐1 and Murf‐1), thereby leading to enhanced muscle breakdown and resulting in muscle atrophy (Lian et al. 2022). Additionally, excessive production of ROS also activates the NF‐κB signaling pathway, triggering inflammatory responses and the release of pro‐inflammatory cytokines, ultimately inducing muscle atrophy (Aquilano et al. 2014).

Currently, effective treatments for sarcopenia in clinical settings remain very limited. Exercise and nutritional interventions, as traditional approaches, are significantly constrained by the physical condition and intestinal absorption capabilities of elderly patients. *Pueraria lobata* , also known as kudzu, is one of the most popular traditional medicines in Asian countries. Puerarin, a major active isoflavone extracted from *pueraria lobata* , possesses various biological activities, including bone protection, anti‐inflammatory, and antioxidant effects (Wang et al. 2020). These activities are highly relevant to conditions like sarcopenia, which is characterized by muscle atrophy and chronic inflammation. Given the established therapeutic effects of puerarin on the skeletal system, particularly its anti‐osteoporotic effects (An et al. 2016; Zhou et al. 2014), and the close physiological relationship between bone and muscle systems, we hypothesized that puerarin could also exert therapeutic effects in sarcopenia. Furthermore, puerarin has been shown to modulate key signaling pathways involved in inflammation and oxidative stress (Ye et al. 2024; Zhou et al. 2022), which are central to the pathogenesis of sarcopenia. However, reports on the application of puerarin in sarcopenia treatment remain scarce. Considering the therapeutic impact of puerarin on osteoporosis, there is speculation regarding its potential efficacy in sarcopenia treatment.

Dexamethasone, a widely used glucocorticoid, has been extensively recognized for inducing a variety of metabolic dysregulations, including imbalances in skeletal muscle protein synthesis and breakdown, when used long‐term or in excess (Oray et al. 2016). One side effect of such medications is the induction of muscle weakness and skeletal muscle atrophy, a phenomenon employed in scientific research to construct experimental models of muscle wasting or atrophy. Dexamethasone, by mimicking a chronic stress state and elevating peripheral glucocorticoid levels, has been confirmed in numerous studies to effectively induce reductions in muscle mass and strength, making it an effective method for constructing models of sarcopenia (Xie et al. 2021; Chiu et al. 2011). This is particularly relevant in the elderly population, where chronic stress states are more pronounced and peripheral glucocorticoid levels are higher, allowing dexamethasone‐induced models to more accurately simulate the pathological state of sarcopenia in older patients (Delivanis et al. 2018). Therefore, in this study, we opted to use dexamethasone‐induced muscle atrophy in C57 mice as a sarcopenia model to assess the potential therapeutic effects of puerarin on sarcopenia. Furthermore, we aimed to investigate the molecular signaling pathways involved to elucidate the mechanisms by which puerarin treats sarcopenia.

## 2 Materials and Methods

### 2.1 Experimental Animal Intervention

In this study, 18 male C57BL/6J mice aged 10 weeks and weighing approximately 20 g were acclimatized in an SPF‐grade animal facility for 1 week before being randomly divided into three experimental groups, with six mice per group. The grouping and treatment methods were as follows: (1) Control group: received daily intraperitoneal injections of sterile PBS in a volume identical to that used for the model group, continuing for 12 days. (2) Sarcopenia model group: administered daily intraperitoneal injections of sterile dexamethasone solution at a dose of 25 mg/kg for 12 consecutive days. Dexamethasone was purchased from Abcam (ab142419), with its concentration and usage duration based on methodologies from previous studies (Shen et al. 2019). (3) Puerarin treatment group: in addition to the daily intraperitoneal injection of 25 mg/kg dexamethasone, similar to the model group, mice were orally administered 150 mg/kg of puerarin solution daily for 12 days. The concentration of puerarin used was based on prior research (Yang, Gao, et al. 2022; Yang, Yang, et al. 2022), acquired from Abcam (abs47000513). In addition, we conducted preliminary in vitro studies using dexamethasone‐induced C2C12 myotubes to evaluate the optimal concentration of puerarin. As shown by crystal violet staining (Figure S1), 1 mM puerarin demonstrated the most effective inhibition of myotube atrophy. Based on the average weight of the mice (20–22 g) and their estimated blood volume (approximately 1.5 mL), we calculated a corresponding oral dose of 150 mg/kg for in vivo application. This dosing aligns with the pharmacological profile of puerarin as documented in the TCMSP database, where its oral bioavailability is reported to be approximately 20%. All experimental procedures strictly adhered to the guidelines approved by the Research Ethics Committee of the School of Life Sciences, Fudan University.

### 2.2 Grip Strength Test

To measure the grip strength of mice, they were placed on the grip strength meter platform (Yiyan Company, Jinan, China). By gently lifting the tail, the body and hind limbs of the mouse were gradually lifted off the platform while the forelimbs naturally grasped the bar. Once the mouse's forelimbs securely gripped the bar, the pulling force on the tail was incrementally increased until the mouse could no longer maintain its hold on the bar. The force displayed on the grip strength meter at the moment the mouse released the bar was recorded. This procedure was repeated three times with a 10‐min rest interval between each test to allow full recovery, and the highest value among the three trials was recorded as the mouse's grip strength measurement.

### 2.3 Rotarod Fatigue Test

The Rotarod test, conducted using a Rotarod apparatus (Calvin Biological Technology Co. Ltd., Nanjing, China), assesses the motor coordination, balance abilities, and fatigue resistance of mice. This method involves observing the mice's ability to maintain balance on a rod that gradually accelerates, indirectly measuring their muscle function and endurance. To minimize stress reactions, mice were first subjected to acclimatization training before the experiment, which included five sessions of constant rotation at 4 rpm (rpm), each lasting 2 min with a 10‐min interval between sessions. Following acclimatization, three test rounds were performed where the rotation speed gradually increased from 4 to 40 rpm, until the mouse fell off the rod or reached the maximum trial duration of 5 min. By recording the duration mice remained on the rod, their motor coordination and resistance to fatigue were evaluated.

### 2.4 Body Composition Analysis and Muscle Sampling

Prior to body composition analysis, each mouse was fasted for 4 h. The body composition analyzer (EchoMRI) was then utilized according to the standard operating procedure to measure fat mass and lean mass. During this process, mice were positioned within the instrument's locator to ensure immobility throughout the measurement. To guarantee data accuracy, this measurement was repeated three times, and the average was calculated to represent the mouse's body composition.

Following the determination of body composition, mice were anesthetized using isoflurane (5% for induction and 1%–2% for maintenance) delivered via a precision vaporizer. Following anesthesia, approximately 1 mL of whole blood was collected into heparinized Eppendorf tubes by enucleation (removal of the eyeball) using toothless forceps. This terminal blood collection method is commonly used in mouse studies where large‐volume samples are required. After allowing the collected blood to stand at room temperature for 2 h, the tubes were centrifuged at high speed. The supernatant plasma was then collected and stored at −80°C. The major muscles of the lower limbs, including the quadriceps, gastrocnemius, anterior tibialis, soleus, and extensor digitorum longus, were extracted using blunt dissection with toothless forceps, meticulously removing any fat adhering to the muscle surface. The weights of these muscles were measured and recorded, with the average weight of the corresponding muscles on both sides representing the actual weight of each muscle. During the sampling process, all collected muscle tissue samples were immediately frozen in liquid nitrogen and subsequently stored at −80°C for future molecular biology experiments. Alternatively, tissue samples were fixed in 4% paraformaldehyde and embedded in paraffin for subsequent histological sectioning and staining analysis.

### 2.5 H&E Staining

The quadriceps muscle paraffin sections were stained utilizing a Hematoxylin and Eosin (H&E) staining kit. Initially, hematoxylin solution was applied to the sections for 5–10 min, targeting the nuclei. Post‐staining, the sections were washed under running water to eliminate excess stain, with a subsequent deionized water rinse for thorough cleansing. For cytoplasmic counterstaining, eosin solution was employed, lasting between 30 s and 3 min. Following staining, a dehydration process was conducted with a series of ethanol washes in ascending concentrations (70%, 80%, 90%, and 100%), each lasting 10 s, and then the sections were cleared in xylene, twice, for 5 min each. After clearance, the sections were mounted using neutral balsam. Microscopic examination of the stained sections revealed nuclei in blue and cytoplasm in shades of pink to red. Clear and intact tissue structures were photographed for documentation.

### 2.6 Immunofluorescence Staining

The process began with dewaxing the paraffin sections, followed by their rehydration through a sequential ethanol series and a final rinse in ultrapure water. Antigen retrieval was then performed in a boiling solution, after which the sections were washed and blocked using 5% goat serum to inhibit non‐specific binding. Overnight incubation with the primary antibody was carried out at 4°C, after which the sections were washed again and incubated with the secondary antibody at room temperature. Details regarding the antibodies utilized are provided in Table S1. The staining procedure was completed by mounting the sections onto slides, preparing them for examination.

### 2.7 Calculation of Muscle Fiber Area

For each muscle specimen, 3–5 photographs from random views were captured to ensure the inclusion of over 150 muscle fibers for area measurement. The images were opened using Adobe Photoshop software by Adobe Inc., USA. The lasso tool was employed to individually outline each muscle fiber, obtaining its area, which was then converted to the actual area of each muscle fiber based on the image's scale bar. The average area was calculated from these 150 muscle fibers, yielding the mean muscle fiber area per mouse. This mean area was utilized to compare differences in muscle fiber size among groups.

### 2.8 Transmission Electron Microscopy Observation

For the electron microscopy study of the gastrocnemius muscle, tissues were quickly excised and sectioned into small pieces, each measuring approximately 2 mm × 2 mm × 2 mm, within 1–3 min. These pieces were immediately fixed in a fresh electron microscopy‐specific fixative and stored at 4°C. The tissues underwent three washes in 0.1 M phosphate buffer (pH 7.4), each lasting 15 min. Subsequently, post‐fixation involved 1% osmium tetroxide in the same buffer for 2 h at room temperature in darkness, followed by another three buffer washes. The dehydration process was carried out at room temperature, using a graduated series of ethanol solutions and acetone. For embedding, the tissue was first infiltrated with a 1:1 mixture of acetone and epoxy resin 812 at 37°C, then with a 1:2 mixture, and finally embedded in pure resin, each step ensuring thorough infiltration. The embedded tissue blocks were polymerized at 60°C for 48 h. Ultrathin sections, ranging from 60 to 80 nm, were then prepared and placed on copper grids. These sections were stained with 2% uranyl acetate and lead citrate, then left to air‐dry overnight. The prepared sections were analyzed and imaged using a transmission electron microscope, providing detailed insights into the ultrastructural characteristics of the muscle tissue.

### 2.9 Plasma ELISA Analysis

The levels of inflammatory cytokines in the plasma of three groups of mice were assessed using the Mouse Inflammatory Cytokine ELISA Kits, following the procedures specified in the ELISA Kit manuals. The ELISA Kits for six inflammatory cytokines were all procured from ELK Biotechnology, with the following sensitivity and detection range details: IL‐1β (sensitivity: 6.4 pg/mL; range: 15.63–1000 pg/mL; catalog: ELK1271), IL‐6 (sensitivity: 3.2 pg/mL; range: 7.82–500 pg/mL; catalog: ELK1157), IL‐10 (sensitivity: 3 pg/mL; range: 7.82–500 pg/mL; catalog: ELK1143), IL‐15 (sensitivity: 6 pg/mL; range: 15.63–1000 pg/mL; catalog: ELK2267), TNF‐α (sensitivity: 6.1 pg/mL; range: 15.63–1000 pg/mL; catalog: ELK1387), and GDF15 (sensitivity: 6.2 pg/mL; range: 15.63–1000 pg/mL; catalog: ELK5776). Additionally, the Mouse GSH ELISA Kit and the Mouse MDA ELISA Kit were used to determine the plasma levels of GSH (sensitivity: 1.25 ng/L; range: 5–120 ng/L; catalog: HB835‐Mu, Shanghai Hengyuan Biotech) and MDA (sensitivity: 0.075 nmol/L; range: 0.3–8 nmol/L; catalog: HB1357‐Mu, Shanghai Hengyuan Biotech), respectively, to assess the oxidative stress levels in the three groups of mice. The experimental procedures were carried out according to the manufacturer's protocols.

### 2.10 Quantitative RT‐PCR Analysis

Total RNA extraction from muscle tissues was carried out using the Trizol method. This process involved lysing the tissues in Trizol reagent (catalog: 15596018CN), separating phases with chloroform, precipitating RNA with isopropanol, and washing with ethanol. The resulting RNA was then dissolved in RNase‐free water. Spectrophotometry at 260 and 280 nm wavelengths was used to assess the concentration and purity of the extracted RNA. Following this, the quantified RNA underwent reverse transcription into cDNA, as per the reverse transcription kit provided by the manufacturer. Quantitative real‐time PCR (qRT‐PCR) analysis was conducted using gene‐specific primers, which are listed in Table S2, in combination with a SYBR Green PCR Master Mix (catalog: RR420A). Target gene expression levels were normalized against the internal reference gene Gapdh, as listed in Table S2. The comparative CT method was utilized for calculating relative gene expression, enabling a precise and quantitative evaluation of gene expression profiles.

### 2.11 Western Blot Analysis

Protein extraction from muscle tissues was achieved through homogenization in a lysis buffer enriched with protease inhibitors, safeguarding against protein degradation. Following centrifugation of the homogenate, the supernatant, rich in extracted proteins, was collected. The bicinchoninic acid (BCA) assay facilitated the determination and normalization of protein concentrations within these extracts. During the immunoblotting procedure, proteins, quantified to equal amounts, underwent separation via SDS‐PAGE and were then transferred to PVDF membranes. To minimize non‐specific binding, these membranes were subjected to blocking and subsequently incubated with primary antibodies specific to the proteins under study. After a series of washes, the membranes received treatment with horseradish peroxidase‐linked secondary antibodies. Visualization of protein bands was accomplished through enhanced chemiluminescence, followed by quantitative assessment via ImageJ software. For comprehensive details on the antibodies employed, refer to Table S1.

### 2.12 Statistical Analysis

In this study, data processing and statistical analyses were conducted using SPSS software (Version 13.0) by IBM Corporation, USA. The normality of data was assessed using theShapiro‐Wilk test. For normally distributed data, statistical significance was evaluated using one‐way ANOVA, while non‐parametric data were analyzed using the Kruskal–Wallis H test. All measurement data presented in graphs are expressed as mean ± standard error (SE), and results were considered statistically significant at a *p* < 0.05.

## 3 Results

### 3.1 Puerarin Prevents Muscle Mass Loss in Dexamethasone‐Induced Sarcopenic Mice

**FIGURE 1:**
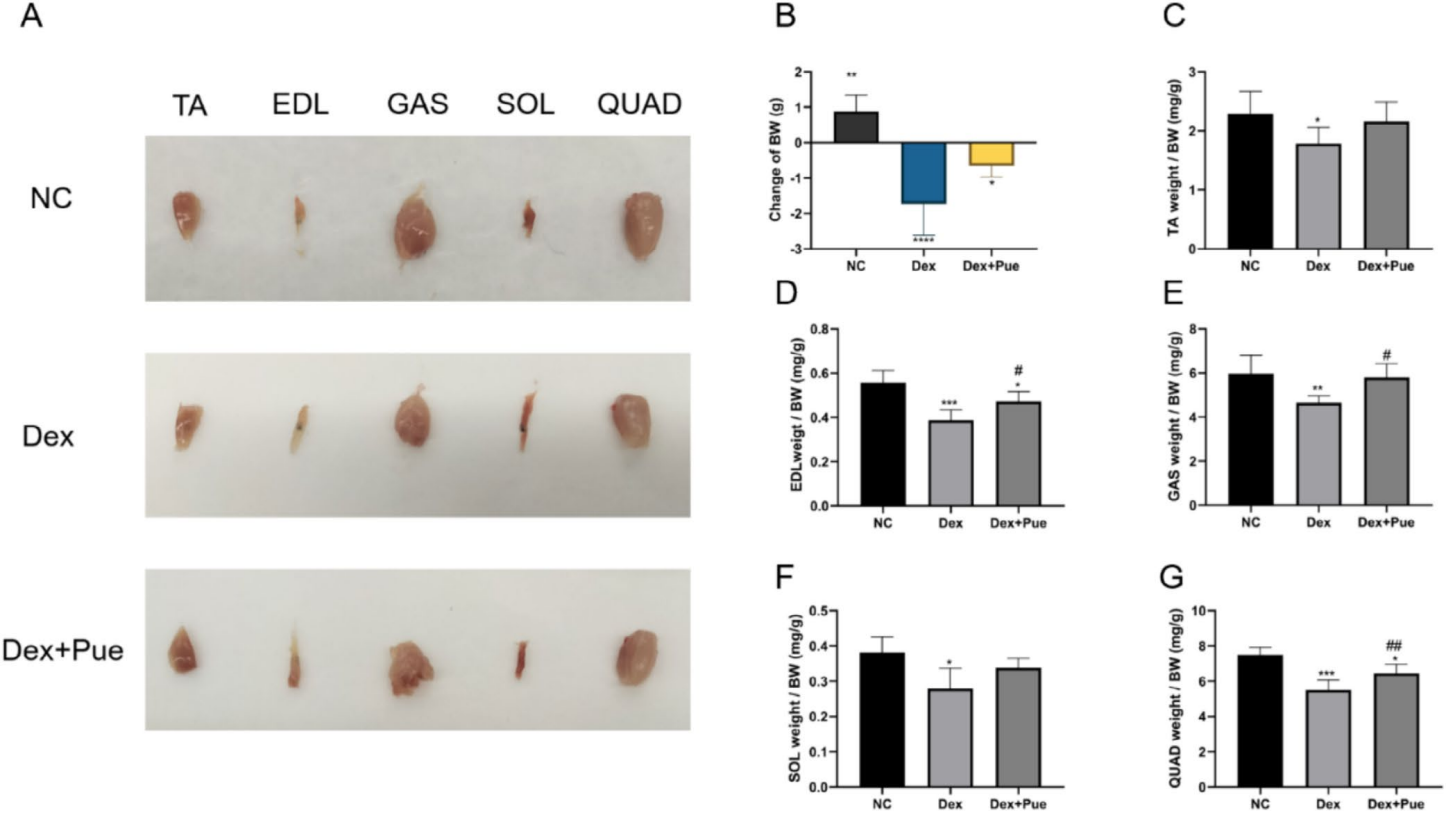
Puerarin prevents muscle mass loss in dexamethasone‐induced sarcopenic mice. (A) Comparative images of muscle specimens from three groups of mice. (B) Body weight changes among the three groups of mice, where BW is an abbreviation for body weight. (C) Comparison of anterior tibialis muscle mass among the three groups, with TA denoting the anterior tibialis. (D) Comparison of extensor digitorum longus muscle mass among the three groups, with EDL denoting the extensor digitorum longus. (E) Comparison of gastrocnemius muscle mass among the three groups, with GAS denoting the gastrocnemius. (F) Comparison of soleus muscle mass among the three groups, with SOL denoting the soleus. (G) Comparison of quadriceps muscle mass among the three groups, with QUAD denoting the quadriceps. *N* = 6, “*” marks statistical significance in comparison with the NC group, *p < 0.05, **p < 0.01, ***p < 0.001; “#” denotes statistical significance compared to the Dex group, ^#^ *p* < 0.05, ^##^ *p* < 0.05. Statistical analysis was performed using one‐way ANOVA for normally distributed data.

Before the experiment commenced, a baseline comparison of body weights among the three groups of mice showed no significant statistical difference, ensuring initial uniformity of the experiment. After 12 days of experimental intervention, the control group exhibited a slight increase in body weight, reflecting a normal growth trend. In contrast, the body weight of mice with dexamethasone‐induced sarcopenia significantly decreased, revealing substantial muscle mass loss and its negative impact on body weight due to dexamethasone. Meanwhile, mice treated with puerarin also showed a trend of weight reduction, but to a lesser extent compared to the dexamethasone group, indicating puerarin's potential to mitigate weight loss caused by dexamethasone. Figure 1A visually demonstrates muscle tissue samples from the three groups, where muscle mass in the puerarin‐treated mice appeared to increase compared to that in the dexamethasone‐treated group. As shown in Figure 1B, puerarin treatment partially reversed the body weight loss induced by dexamethasone. Further comparison of key lower limb muscle mass differences among the three groups (Figure 1C–G) showed that all muscle groups' weights significantly decreased in dexamethasone‐induced sarcopenic mice compared to the control group, further corroborating dexamethasone's role in promoting muscle mass loss. However, in the puerarin treatment group, despite reductions in the weight of the extensor digitorum longus and quadriceps muscles compared to the control group, this reduction was significantly mitigated compared to mice treated only with dexamethasone, especially in the extensor digitorum longus, gastrocnemius, and quadriceps muscles, where puerarin treatment significantly improved muscle mass.

### 3.2 Puerarin Mitigates the Decline in Lean Body Mass and Loss of Muscle Function in Dexamethasone‐Induced Sarcopenic Mice

**FIGURE 2:**
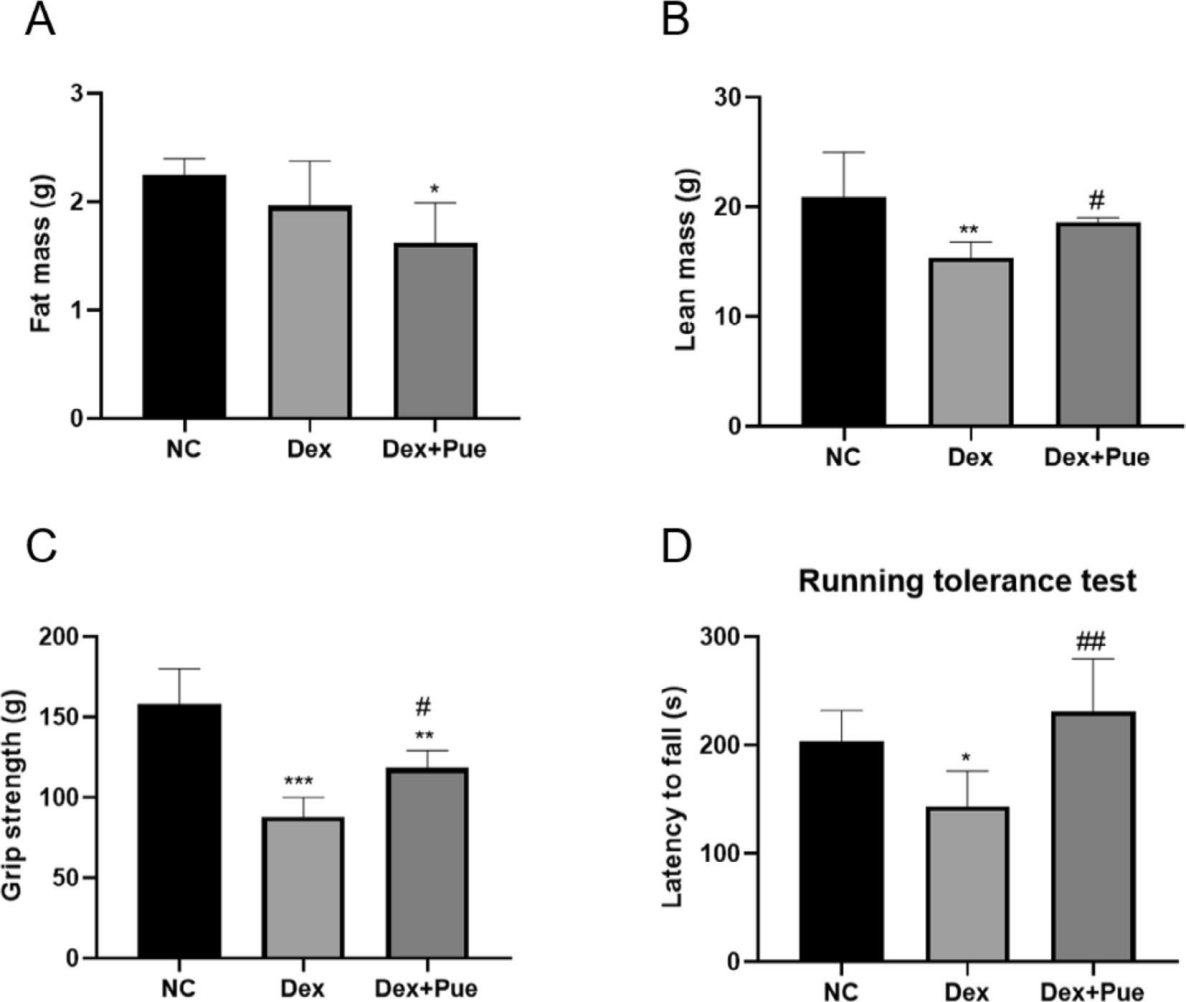
Puerarin mitigates the decline in lean body mass and loss of muscle function in dexamethasone‐induced sarcopenic mice. (A) Comparison of fat body mass across three groups of mice; (B) Comparison of lean body mass across three groups of mice. (C) Comparison of grip strength across three groups of mice; (D) Comparison of the duration on the rotarod in the rotarod test among the three groups of mice. *N* = 6, “*” signifies statistical significance compared to the NC group, *p < 0.05, **p < 0.01, ***p < 0.001; “#” indicates statistical significance compared to the Dex group, ^#^ *p* < 0.05, ^##^ *p* < 0.05. Statistical analysis was performed using Kruskal‐Wallis H test for non‐normally distributed data.

Body composition analysis revealed that mice treated with puerarin exhibited a significant decrease in fat mass compared to the control group (*p* < 0.05), whereas the fat mass of mice in the dexamethasone model group was reduced compared to the control group, but the difference was not statistically significant (Figure 2A). Additionally, comparisons of lean body mass showed that the control group's lean body mass was significantly higher than that of the two groups treated with dexamethasone (Figure 2B). Notably, the lean body mass of the puerarin treatment group was significantly increased compared to the sarcopenia model group (*p* < 0.05).

**FIGURE 3:**
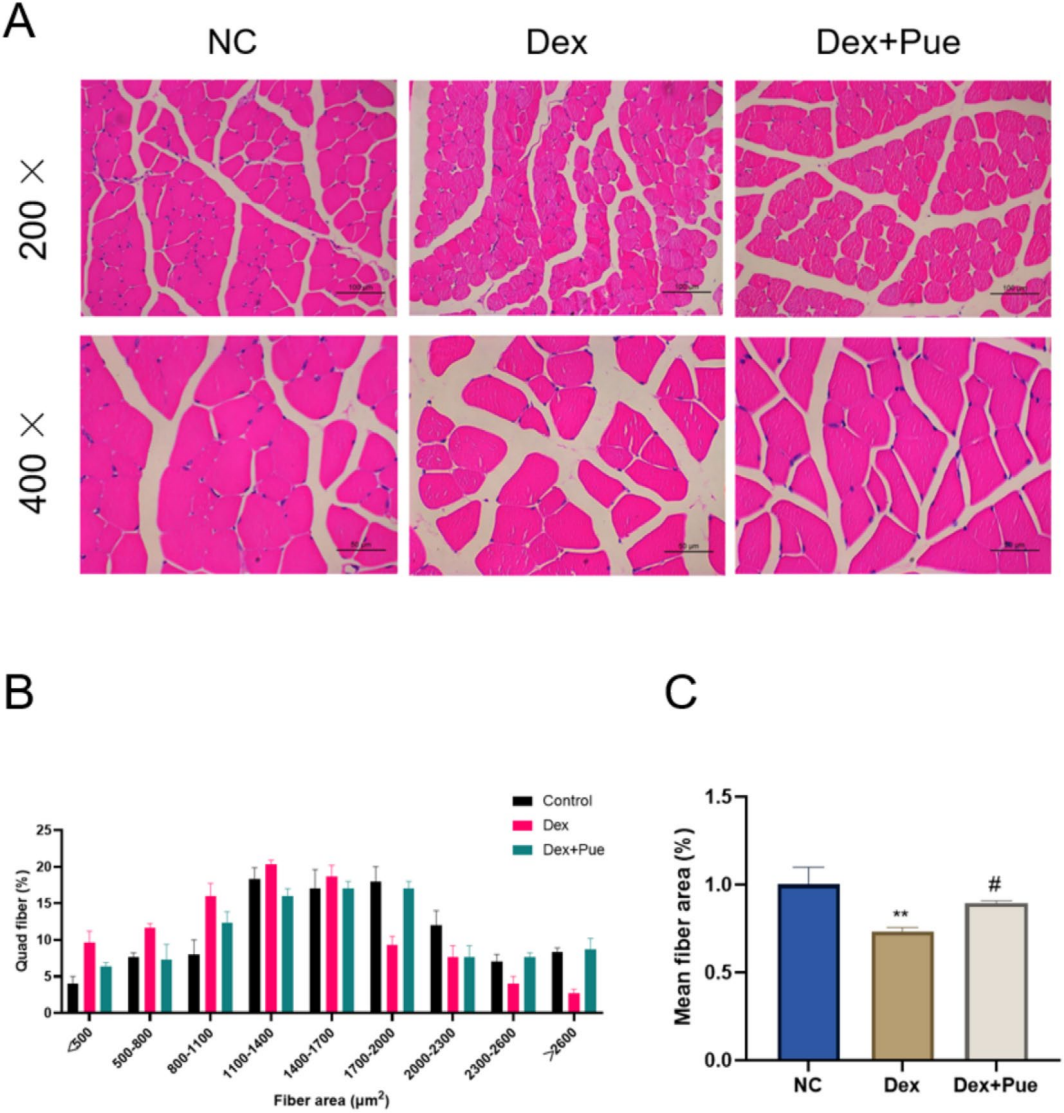
Puerarin inhibits the reduction of muscle fiber area in dexamethasone‐induced sarcopenic mice. (A) H&E staining results of the quadriceps muscle from different groups of mice. (B) Distribution diagram of muscle fiber area in the quadriceps among the three groups of mice. (C) Comparison of the average muscle fiber area in the quadriceps among the three groups of mice. *N* = 4, “*” indicates statistical significance compared with the NC group, **p < 0.01; “#” denotes statistical significance compared to the Dex group, ^#^ *p* < 0.05. Statistical analysis was performed using one‐way ANOVA for normally distributed data.

Muscle function comparisons among the three groups of mice, as shown in Figure 3C,D, indicated that dexamethasone intervention significantly reduced grip strength in mice, reflecting the induced muscle function decline. In contrast, mice that received puerarin treatment demonstrated relatively improved muscle strength, with significantly higher grip strength than mice treated with dexamethasone alone (*p* < 0.05). In the rotarod fatigue test, the endurance of mice in the puerarin treatment group, as measured by the time spent on the rod, was significantly longer than that of mice treated only with dexamethasone (*p* < 0.01).

### 3.3 Puerarin Inhibits the Reduction of Muscle Fiber Area in Dexamethasone‐Induced Sarcopenic Mice

In this study, to explore the effects of dexamethasone and puerarin on skeletal muscle fiber size, we employed H&E staining for histological observation of the quadriceps muscle cross‐sections. H&E staining results (Figure 3A) revealed a significant reduction in the cross‐sectional area of muscle fibers in mice treated with dexamethasone compared to the untreated control group. Quantitative morphological analysis of the muscle fiber cross‐sectional area showed a decrease in the number of smaller fibers and an increase in the number of larger fibers in the puerarin treatment group (Figure 3B). Consequently, the average cross‐sectional area of muscle fibers in mice treated with puerarin was significantly larger than that in the model group treated only with dexamethasone (Figure 3C). These findings indicate that puerarin possesses a marked anti‐atrophic effect, effectively countering the reduction in muscle fiber size induced by dexamethasone.

### 3.4 Puerarin Reduces the Expression of Muscle Atrophy Markers in Dexamethasone‐Induced Sarcopenic Mice

**FIGURE 4:**
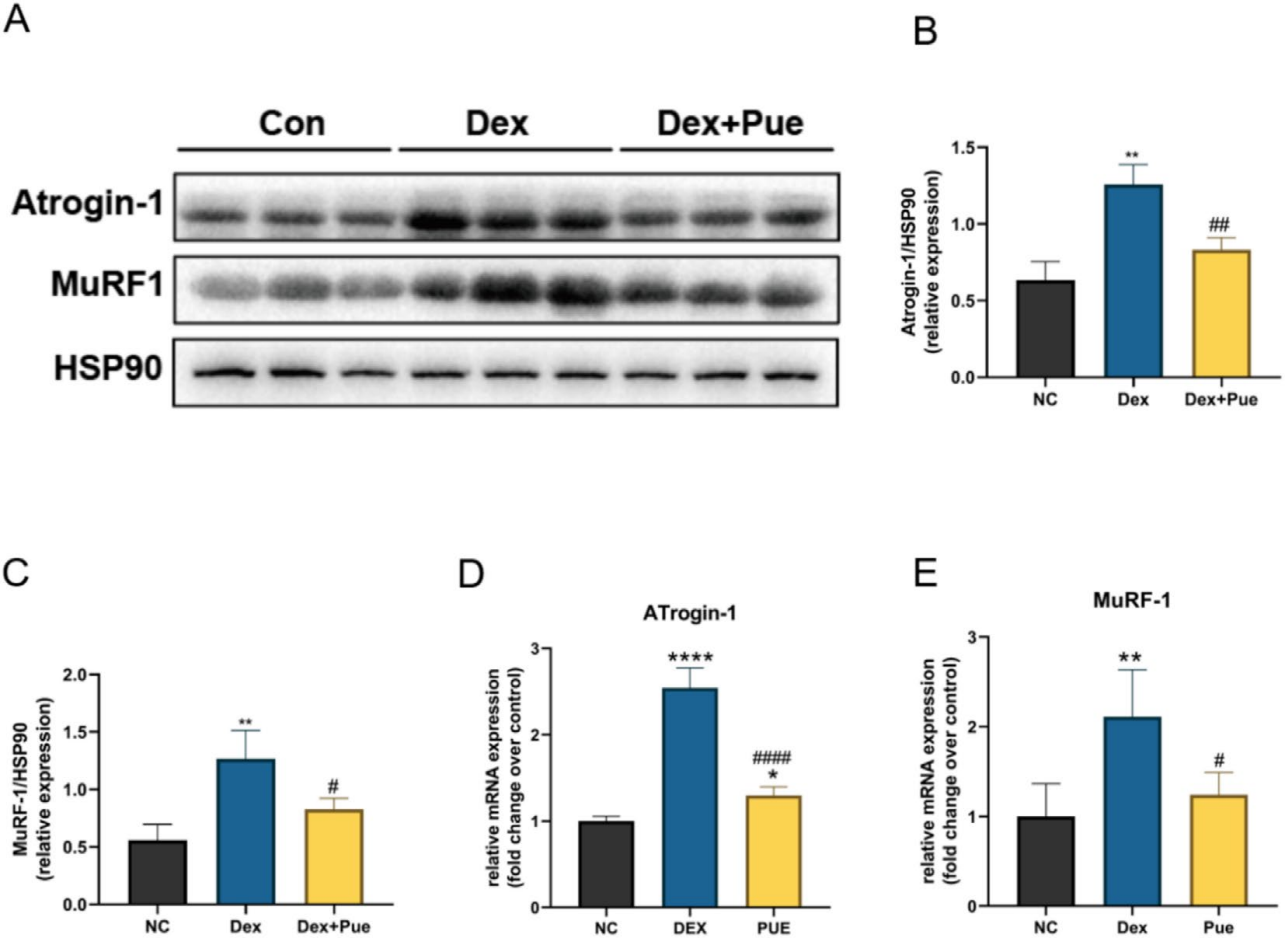
Puerarin reduces the expression of muscle atrophy markers in dexamethasone‐induced sarcopenic mice. (A) Expression levels of atrophy marker proteins in the quadriceps muscle of three groups of mice at the protein level. (B) Statistical diagram of grayscale values differences for Atrogin‐1 protein among the three groups. (C) Statistical diagram of grayscale values differences for MuRF‐1 protein among the three groups. (D) Relative mRNA expression levels of Atrogin‐1 in the quadriceps muscle among the three groups. (E) Relative mRNA expression levels of MuRF‐1 in the quadriceps muscle among the three groups. *N* = 3, “*” indicates statistical significance compared with the NC group, *p < 0.05, **p < 0.01, ****p < 0.0001; “#” denotes statistical significance compared to the Dex group, ^#^ *p* < 0.05, ^##^ *p* < 0.01, ^####^ *p* < 0.0001. Statistical analysis was performed using one‐way ANOVA for normally distributed data.

The ubiquitin ligases E3, Atrogin‐1, and MuRF‐1 are widely regarded as key molecular markers in the process of skeletal muscle atrophy (Gumucio and Mendias 2013). Various animal models, including denervation, immobilization, and dexamethasone‐induced skeletal muscle atrophy models, have reported upregulation of these two genes' expression levels (Takase et al. 2021; Doss et al. 2022). Under conditions of sarcopenia, the increased expression of muscle atrophy‐related proteins reflects the activation of the protein degradation pathway. As shown in Figure 4A, in mice with dexamethasone‐induced sarcopenia, the expression levels of the muscle atrophy‐associated proteins Atrogin‐1 and MuRF‐1 were significantly higher than in the control group. This indicates that dexamethasone induced the activation of the protein degradation pathway in skeletal muscle tissue, promoting muscle atrophy. However, when mice treated with dexamethasone also received puerarin intervention, the upregulation of Atrogin‐1 and MuRF‐1 protein expression was significantly inhibited (Figure 4B,C, *p* < 0.05). These results suggest that puerarin can reduce the expression of muscle atrophy‐associated marker proteins at the protein level, thereby inhibiting muscle atrophy triggered by dexamethasone. Importantly, the trends observed at the mRNA level were consistent with those at the protein level for both Atrogin‐1 and MuRF‐1 (Figure 4D,E), suggesting that the transcriptional regulation of these muscle atrophy markers is closely mirrored at the translational level. This indicates that puerarin may exert its protective effect by downregulating both the gene and protein expression of key components of the ubiquitin–proteasome degradation pathway.

### 3.5 Puerarin Promotes the Transition From Slow to Fast Muscle Fibers in Dexamethasone‐Induced Sarcopenic Mice

**FIGURE 5:**
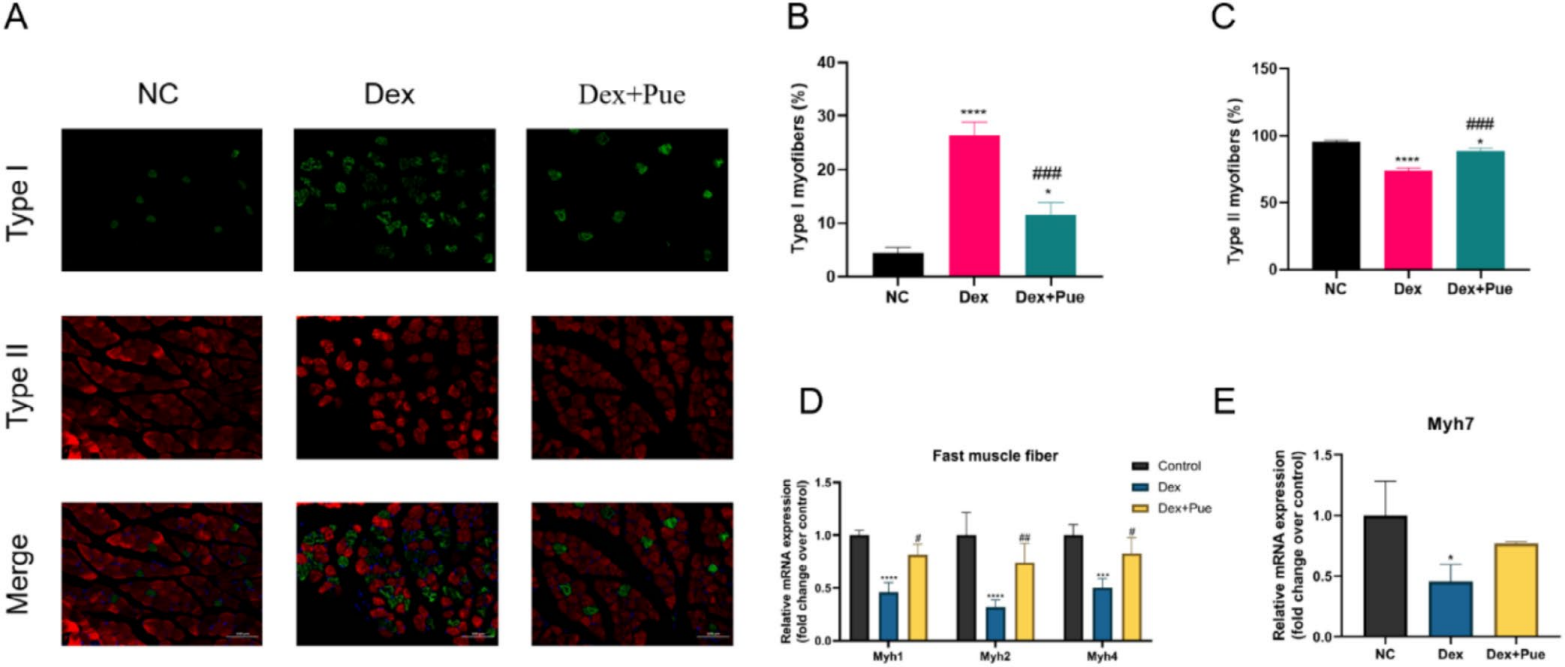
Puerarin promotes the transition from slow to fast muscle fibers in dexamethasone‐induced sarcopenic mice. (A) Immunofluorescence staining results for the gastrocnemius muscle across different mouse groups. (B) Proportion of Type I fibers in the gastrocnemius muscle among the three groups of mice. (C) Proportion of Type II fibers in the gastrocnemius muscle among the three groups of mice. (D) Comparison of mRNA expression levels of fast‐twitch fiber genes Myh1, Myh2, and Myh4 in the gastrocnemius muscle among the three groups. (E) Comparison of mRNA expression levels of the slow‐twitch fiber gene Myh7 in the gastrocnemius muscle among the three groups. *N* = 4, “*” indicates statistical significance compared with the NC group, *p < 0.05, **p < 0.01, ****p < 0.0001; “#” denotes statistical significance compared to the Dex group, ^#^ *p* < 0.05, ^##^ *p* < 0.01, ^###^ *p* < 0.001, ^####^ *p* < 0.0001. Statistical analysis was performed using one‐way ANOVA for normally distributed data.

To delve into the impact of puerarin on skeletal muscle fiber type transformation, particularly changes induced by dexamethasone, we analyzed the gastrocnemius muscle (a mixed muscle containing both slow and fast fibers) of three groups of mice using immunofluorescence staining. The results (Figure 5A) demonstrated that in the two groups of mice treated with dexamethasone, the proportion of fast fibers significantly decreased while that of slow fibers correspondingly increased. This indicates that dexamethasone induced a shift from fast, glycolytic, fatigue‐prone fibers to endurance‐oriented, oxidative metabolism fibers. However, this dexamethasone‐induced fiber type transition was effectively mitigated with puerarin intervention, suggesting that puerarin may counteract muscle atrophy induced by dexamethasone by maintaining the proportion of fast fibers. Quantitative analysis of immunofluorescence staining (Figure 5B,C) also revealed a significant increase in the proportion of Type II fibers in the puerarin‐treated group compared to the dexamethasone group (*p* < 0.001). Furthermore, we assessed the expression changes of Myh1, Myh2, Myh4, and Myh7 in the gastrocnemius muscle of the three groups of mice. Specifically, Myh7 encodes myosin heavy chain primarily found in fatigue‐resistant, slow‐twitch Type I fibers (Queeno et al. 2023); Myh2 encodes myosin heavy chain IIa present in fast‐twitch but relatively fatigue‐resistant Type IIa fibers, while Myh1 and Myh4 encode myosin heavy chains IIx and IIb, respectively, found in fast‐twitch, easily fatigued Type IIx and Type IIb fibers, which can rapidly generate force but have lower endurance (Liu et al. 2021). The results (Figure 5D,E) indicated that compared to the dexamethasone‐induced sarcopenic mice, the puerarin‐treated group showed significant changes in muscle fiber types, especially an increase in the expression of fast‐twitch fibers (Myh1, Myh2, Myh4) at a rate significantly higher than the increase in slow‐twitch fibers (Myh7).

### 3.6 Puerarin Reduces Skeletal Muscle Cell Apoptosis in Dexamethasone‐Induced Sarcopenic Mice

**FIGURE 6:**
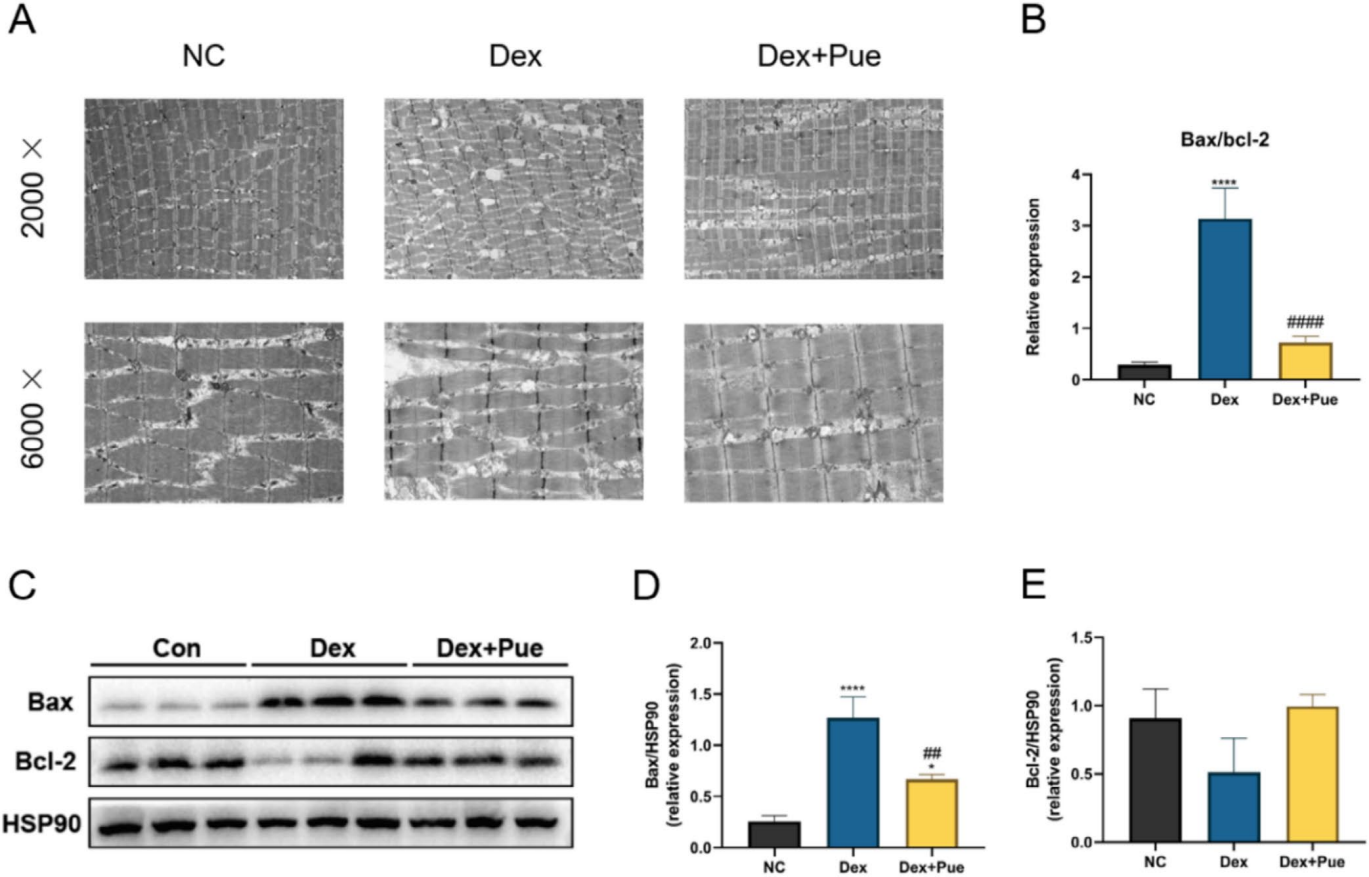
Puerarin Reduces Skeletal Muscle Cell Apoptosis in Dexamethasone‐Induced Sarcopenic Mice. (A) Electron microscopy results show that puerarin effectively reduces the destruction of muscle microstructure in the dexamethasone‐induced sarcopenia mouse model. (B) Differences in the Bax/Bcl‐2 ratio among the three groups of mice. (C) Expression levels of apoptotic proteins at the protein level in the quadriceps muscle among the three groups of mice. (D) Statistical diagram of grayscale value differences for Bax protein among the three groups. (E) Statistical diagram of grayscale value differences for Bcl‐2 protein among the three groups. *N* = 3, “*” denotes statistical significance compared with the NC group, *p < 0.05, ***p < 0.001, ****p < 0.0001; “#” indicates statistical significance compared to the Dex group, ^##^ *p* < 0.01, ^####^ *p* < 0.0001. Statistical analysis was performed using one‐way ANOVA for normally distributed data.

To investigate the ultrastructural changes in muscle fibers, we meticulously examined the microstructural alterations in the gastrocnemius muscle using TEM. As depicted in Figure 6A, muscle fibers in the control group exhibited a highly organized structure with a clear and regular pattern of sarcomeres, demonstrating typical characteristics of healthy muscle tissue. In contrast, muscle fibers in dexamethasone‐induced sarcopenic mice displayed significant disarray, irregular spacing between sarcomeres, and abnormal mitochondrial morphology. Muscle fibers in the puerarin‐treated group, however, showed a more regular arrangement and more intact mitochondrial morphology. This suggests that puerarin may effectively inhibit the muscle structural damage caused by dexamethasone. We further evaluated the expression levels of apoptosis‐related proteins Bax and Bcl‐2. Protein expression analysis revealed a significant upregulation of Bax and downregulation of Bcl‐2 in dexamethasone‐induced sarcopenic mice, leading to a Bax/Bcl‐2 ratio significantly higher than that in the control and puerarin‐treated groups (Figure 6B). The significant increase in this ratio in dexamethasone‐induced sarcopenic mice reveals the exacerbated apoptotic process induced by dexamethasone, whereas puerarin treatment effectively modulated this balance, lowering the Bax/Bcl‐2 ratio. This indicates the potential of puerarin to protect muscle fibers from damage induced by dexamethasone.

### 3.7 Puerarin Modulates the Expression of Inflammatory and Antioxidant Factors in Dexamethasone‐Induced Sarcopenic Mice

**FIGURE 7:**
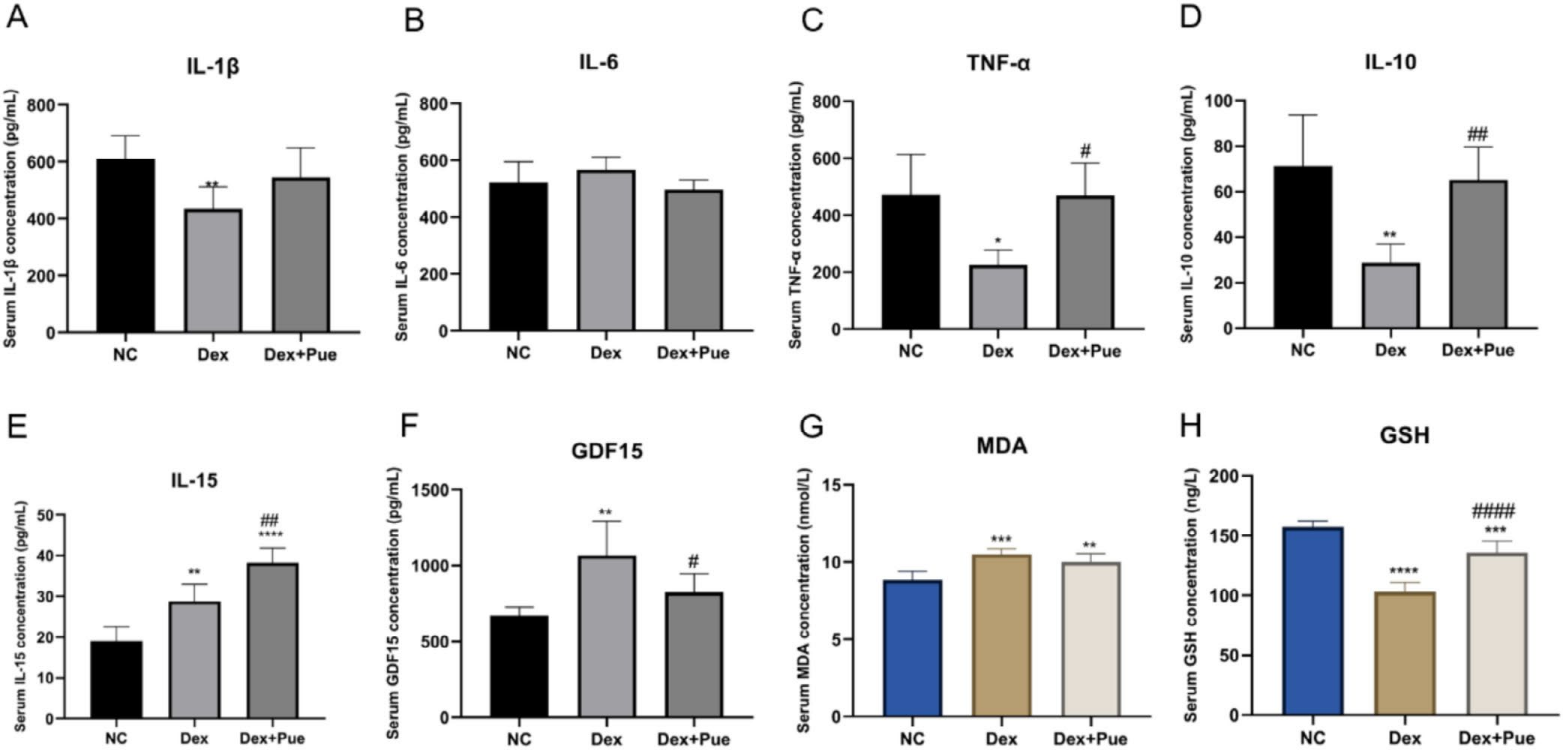
Puerarin Modulates the Expression of Inflammatory and Antioxidant Factors in Dexamethasone‐Induced Sarcopenic Mice. (A) Comparison of serum IL‐1β levels among the three groups of mice. (B) Comparison of serum IL‐6 levels among the three groups of mice. (C) Comparison of serum TNF‐α levels among the three groups of mice. (D) Comparison of serum IL‐10 levels among the three groups of mice. (E) Comparison of serum IL‐15 levels among the three groups of mice. (F) Comparison of serum GDF15 levels among the three groups of mice. (G) Comparison of serum MDA levels among the three groups of mice. (H) Comparison of serum GSH levels among the three groups of mice. *N* = 6, “*” indicates statistical significance compared with the NC group, *p < 0.05, **p < 0.01, ****p < 0.0001; “#” denotes statistical significance compared to the Dex group, ^#^ *p* < 0.05, ^##^ *p* < 0.01. Statistical analysis was performed using one‐way ANOVA for normally distributed data (IL‐6, IL‐15, MDA, and GSH) and the Kruskal‐Wallis H test for non‐normally distributed data (IL‐1β, TNF‐α, IL‐10, and GDF15).

To investigate the effect of puerarin on the chronic inflammatory state induced by dexamethasone in sarcopenic mice, we measured the serum levels of six inflammation‐related cytokines (IL‐1β, TNF‐α, IL‐10, IL‐6, IL‐15, and GDF15). As shown in Figure 7A–F, levels of IL‐1β and TNF‐α were significantly decreased in dexamethasone‐induced sarcopenic mice compared to the control group, reflecting the anti‐inflammatory effect of dexamethasone. Concurrently, the decrease in IL‐10 levels suggested potential impairment of immune system function caused by dexamethasone. In the puerarin treatment group, levels of IL‐1β, TNF‐α, and IL‐10 showed a tendency to recover compared to the dexamethasone‐induced sarcopenic mice, with changes in TNF‐α and IL‐10 reaching statistical significance (*p* < 0.05). Additionally, levels of IL‐6, IL‐15, and GDF15 increased in dexamethasone‐induced sarcopenic mice relative to the control group, particularly with a significant rise in GDF15 levels (*p* < 0.01). After puerarin treatment, levels of IL‐6 and GDF15 decreased relative to the dexamethasone group. In contrast, IL‐15 levels significantly increased in the puerarin treatment group (*p* < 0.01).

Moreover, we measured the levels of oxidative stress markers GSH and MDA in the serum of the three groups of mice. Compared to the control group, the dexamethasone‐treated groups showed significantly higher levels of MDA and significantly lower levels of GSH, indicating a clear state of oxidative stress and damage to the antioxidant defense system induced by dexamethasone. Mice treated with puerarin exhibited an opposite trend, with significantly increased serum GSH levels and decreased MDA levels. These changes suggest that puerarin may enhance antioxidant defense capacity and reduce the formation of lipid peroxidation products, thereby alleviating oxidative stress damage induced by dexamethasone.

### 3.8 Puerarin Delays Muscle Atrophy Development in Dexamethasone‐Induced Sarcopenic Mice by Inhibiting the TNF‐α/NF‐κB Pathway

**FIGURE 8:**
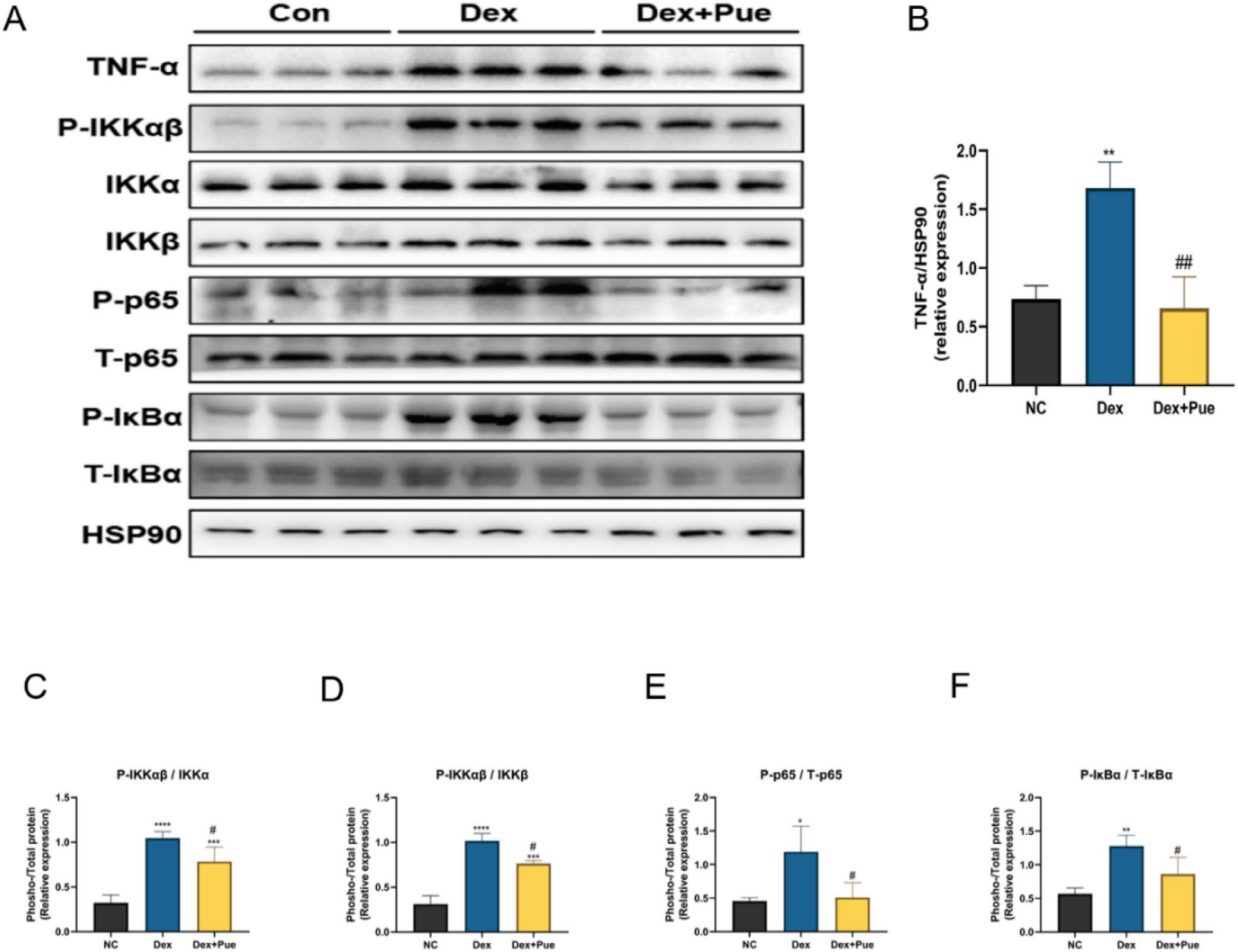
Puerarin reduces muscle atrophy in mice with dexamethasone‐induced sarcopenia by inhibiting the TNF‐α/NF‐kB pathway. (A) Expression levels of proteins in the TNF‐α/NF‐kB signaling pathway in the quadriceps muscle among the three groups of mice at the protein level. (B) Statistical diagram of grayscale value differences for TNF‐α protein in the muscles among the three groups. (C) Statistical diagram of relative grayscale value differences for phosphorylated Ikkα protein among the three groups. (D) Statistical diagram of relative grayscale value differences for phosphorylated Ikkβ protein among the three groups. (E) Statistical diagram of relative grayscale value differences for phosphorylated P65 protein among the three groups. (F) Statistical diagram of relative grayscale value differences for phosphorylated IκBα protein among the three groups. *N* = 3, “*” indicates statistical significance compared with the NC group, *p < 0.05, **p < 0.01, ***p < 0.001, ****p < 0.0001; “#” denotes statistical significance compared to the Dex group, ^#^ *p* < 0.05, ^##^ *p* < 0.01. Statistical analysis was performed using one‐way ANOVA for normally distributed data.

The TNF‐α/NF‐κB signaling pathway plays a crucial role in cell‐mediated immunity and inflammatory responses, regulating various biological processes including cell survival, proliferation, differentiation, and apoptosis (Yang, Gao, et al. 2022; Yang, Yang, et al. 2022). In the pathogenesis of sarcopenia, the upregulation of TNF‐α and activation of the NF‐κB pathway are closely associated with muscle atrophy. This is achieved by promoting the expression of muscle atrophy‐related genes such as Atrogin‐1 and MuRF‐1, accelerating protein degradation and muscle atrophy (Reid and Li 2001). Therefore, we examined the expression levels of core proteins in the TNF‐α/NF‐κB signaling pathway to elucidate the mechanism by which puerarin delays the development of muscle atrophy in dexamethasone‐induced sarcopenic mice. As shown in Figure 8, in mice with dexamethasone‐induced sarcopenia, the expression level of TNF‐α protein in muscle was significantly increased compared to the control group. However, this increase was notably inhibited under puerarin intervention. Additionally, in the muscles of dexamethasone‐induced sarcopenic mice, the phosphorylation levels of Ikkα, Ikkβ, P65, and IκBα were significantly higher than those in the control group. After treatment with puerarin, the phosphorylation levels of these proteins were significantly reduced. This indicates that puerarin can inhibit the activation of the TNF‐α/NF‐κB signaling pathway by reducing TNF‐α levels and downregulating the phosphorylation of Ikkα, Ikkβ, P65, and IκBα. This further decreases the activation of the ubiquitin‐proteasome pathway, inhibits the transcriptional activity of Atrogin‐1 and MuRF‐1 in muscle, thereby effectively delaying muscle atrophy caused by dexamethasone.

## 4 Discussion

Skeletal muscle plays an essential role in maintaining body movement, energy metabolism balance, and regulating the release of various physiological factors (Karstoft and Pedersen 2016). Significant loss of muscle mass not only poses a threat to an individual's functional status but may also increase the risk of diseases and reduce survival rates (Sartori et al. 2021). Although exercise training and nutritional supplementation are the primary strategies for combating sarcopenia, their clinical efficacy remains limited, necessitating the development of new therapeutic approaches to more effectively counter muscle atrophy. Against this backdrop, puerarin, a natural compound with anti‐inflammatory and antioxidant properties, has been explored for its therapeutic application in treating diseases such as osteoporosis (An et al. 2016). Considering the close physiological and pathological connections between muscle and bone, we hypothesize that puerarin may also exert a therapeutic effect in muscle diseases, especially in sarcopenia.

Skeletal muscle tissue constitutes approximately half of the total body mass in mice, with the diminution of muscle mass exerting a direct influence on the aggregate body weight of the organism (Wang and Pessin 2013). In this study, mice with dexamethasone‐induced sarcopenia exhibited a significant decrease in body weight, a phenomenon that was mitigated following intervention with puerarin, indicating puerarin's potential therapeutic effect against muscle mass loss caused by dexamethasone. Detailed muscle tissue measurements further revealed that dexamethasone intervention significantly reduced the mass of key muscles including the anterior tibialis, gastrocnemius, soleus, extensor digitorum longus, and quadriceps, whereas puerarin intervention reversed this loss of mass, especially in the extensor digitorum longus, gastrocnemius, and quadriceps, suggesting its role in enhancing muscle mass. Moreover, body composition analysis showed that puerarin could reduce fat mass while increasing lean body mass, reflecting its dual benefits in regulating body fat and protecting lean body mass. Muscle function assessment demonstrated that puerarin treatment significantly improved grip strength in mice with dexamethasone‐induced sarcopenia, further confirming puerarin's positive effects on muscle function. However, grip strength measurements were conducted only once at the end of the modeling period and not at multiple time points throughout the experiment. This was to avoid frequent measurements that could induce stress in mice, potentially affecting the accuracy of experimental outcomes. Additionally, mice in the puerarin treatment group also exhibited significantly improved endurance in the rotarod fatigue test, reflecting puerarin's potential positive impact on muscle function and endurance. Surprisingly, the performance of mice in the puerarin group in the rotarod fatigue test even surpassed that of the control group, although the difference did not reach statistical significance, possibly due to sample size limitations or variability in experimental conditions.

The functionality and adaptability of skeletal muscle largely depend on its fiber composition, which can be classified into slow and fast fibers based on their contraction speed and metabolic characteristics. In the lower limb muscle groups of mice, the composition of muscle fibers varies among different muscles. For instance, the soleus muscle is predominantly made up of endurance‐type slow fibers, the quadriceps consist largely of fast fibers, and both the gastrocnemius and anterior tibialis muscles feature a mix of slow and fast fibers (Tyagi et al. 2017). This compositional diversity renders the gastrocnemius muscle an ideal subject for studying fiber type transitions, as it allows for the observation of transitions between slow and fast fibers. In this study, we utilized immunofluorescence staining and gene expression analysis to thoroughly investigate the effect of puerarin on fiber type transitions within the gastrocnemius muscle. In mice with dexamethasone‐induced sarcopenia, we observed a significant reduction in the average cross‐sectional area of muscle fibers, particularly in the reduction of fast fibers, leading to a shift in muscle type from fast to slow. This phenomenon, akin to the muscle atrophy process induced by aging (Fappi et al. 2019), suggests that dexamethasone may induce muscle atrophy by mimicking changes in muscle during the aging process. However, puerarin treatment effectively inhibited this process, not only restoring the average cross‐sectional area of muscle fibers but also increasing the number of larger fibers. These changes indicate the potential of puerarin to protect fast fibers and promote muscle mass recovery, thereby counteracting muscle atrophy induced by dexamethasone.

Mitochondrial dysfunction can lead to excessive production of ROS and loss of mitochondrial membrane potential, further triggering apoptotic signaling pathways and accelerating the process of muscle atrophy (de Oliveira et al. 2015). Apoptosis, or programmed cell death, is an orderly and controlled process of cellular self‐destruction, essential for maintaining tissue health and stability (Xu et al. 2019). However, in disease states such as sarcopenia, abnormal activation of apoptosis leads to a reduction in muscle fiber numbers, consequently causing a decline in muscle mass and function (Cheema et al. 2015). The regulation of apoptosis involves multiple signaling pathways, among which the balance between Bax and Bcl‐2 plays a decisive role in the initiation and inhibition of the apoptotic process (King et al. 2023). Bax promotes apoptosis, while Bcl‐2 prevents it, and maintaining a balance between these two is crucial for the survival of muscle cells. In our study, observations from TEM images revealed mitochondrial swelling and destruction in the gastrocnemius muscle of dexamethasone‐induced sarcopenic mice, highlighting the role of mitochondrial dysfunction and apoptosis in the development of sarcopenia. TEM images after puerarin treatment showed a more regular arrangement of muscle fibers and improvement in sarcomere patterns, suggesting a potential cytoprotective effect of puerarin, mitigating cellular structural damage caused by dexamethasone. Results from the expression of apoptotic proteins further elucidated the potential mechanism by which puerarin exerts an anti‐apoptotic effect, indicating that puerarin could mitigate mitochondrial impairment and dexamethasone‐induced apoptosis. This protective action on muscle cells suggests a strategic intervention by puerarin in preserving cellular integrity, thereby decelerating the progression of muscle atrophy.

In inflammation and immune responses, cytokines play a pivotal regulatory role. Our findings indicated that dexamethasone intervention significantly decreased the levels of IL‐1β and TNF‐α, concurrently causing a reduction in IL‐10 levels. This reflects the anti‐inflammatory effects of dexamethasone and its potential implications for compromising immune system functionality. In contrast, treatment with puerarin elicited a rebound in the levels of IL‐1β, TNF‐α, and IL‐10, with particularly notable increases in TNF‐α and IL‐10, unveiling puerarin's potential positive role in modulating immune responses and alleviating immunosuppression induced by dexamethasone. Additionally, dexamethasone intervention was observed to elevate the levels of IL‐6, IL‐15, and GDF15, especially marking a significant rise in GDF15, which suggests a state of chronic stress and inflammation induced by dexamethasone. Puerarin treatment resulted in a relative decrease in the levels of IL‐6 and GDF15, with a significant reduction in GDF15, hinting at puerarin's potential effectiveness in mitigating chronic inflammation and stress conditions. Nevertheless, the pronounced elevation of IL‐15 levels observed in the group treated with puerarin may reflect the multifaceted role of puerarin in the regulation of immune responses, potentially through the augmentation of specific immune cell functions, which in turn could facilitate enhanced immune surveillance and tissue repair mechanisms within the organism (O'Leary et al. 2017).

In the present study, dexamethasone‐treated mice exhibited significantly higher levels of MDA and significantly lower levels of GSH (Figure 7G,H), suggesting a state of systemic oxidative stress. The oxidative stress markers MDA and GSH are well‐established indicators of cellular damage caused by ROS. ROS are known to accumulate in skeletal muscle during muscle‐wasting conditions, leading to protein oxidation, degradation of muscle fibers, and ultimately muscle atrophy (de Oliveira et al. 2015). MDA, a product of lipid peroxidation, is a key indicator of oxidative damage, while GSH, an important antioxidant, plays a protective role by neutralizing ROS. This increase in MDA is particularly concerning because lipid peroxidation can lead to further cellular damage, including mitochondrial dysfunction, which is a hallmark of sarcopenia (Lian et al. 2022). In contrast, puerarin treatment significantly reversed the oxidative stress induced by dexamethasone, as evidenced by the reduction in MDA levels and the increase in GSH levels. This suggests that puerarin not only exerts direct antioxidant effects but also upregulates endogenous antioxidant defense mechanisms, thereby mitigating the progression of oxidative damage.

The TNF‐α/NF‐κB signaling pathway plays a significant role in the onset and progression of sarcopenia. In individuals with sarcopenia, the expression levels of TNF‐α typically rise, activating the NF‐κB inflammatory pathway, leading to an increase in the release of pro‐inflammatory cytokines, exacerbating muscle inflammation and atrophy. Additionally, the activation of the NF‐κB signaling pathway can stimulate the ubiquitin‐proteasome system to increase the degradation of muscle proteins and inhibit the mTOR signaling pathway, preventing the synthesis of muscle proteins, ultimately resulting in muscle atrophy (Chen et al. 2016). Our results demonstrated that in mice with dexamethasone‐induced sarcopenia, the expression of TNF‐α protein significantly increased, along with a notable elevation in the phosphorylation levels of Ikkα, Ikkβ, P65, and IκBα, revealing the critical role of the TNF‐α/NF‐κB signaling pathway in the development of sarcopenia. However, when mice with dexamethasone‐induced sarcopenia were treated with puerarin, the expression levels and phosphorylation states of these proteins were significantly decreased, indicating that puerarin can effectively inhibit the activation of the TNF‐α/NF‐κB signaling pathway. This finding suggests that puerarin may decelerate the progression of muscle atrophy through modulating the TNF‐α/NF‐κB signaling pathway mechanism.

Besides, this study has several limitations. First, sarcopenia was modeled using dexamethasone‐induced muscle atrophy, which may not fully represent the multifactorial nature of age‐related sarcopenia. Therefore, future studies are needed to validate the efficacy of puerarin in naturally aged animal models and to explore its potential synergistic effects when combined with other therapeutic strategies such as exercise or dietary interventions. Another limitation of this study is that oxidative stress markers were assessed in serum rather than directly in muscle tissue, due to sample volume constraints. We recognize that measuring these markers in muscle would more accurately reflect local oxidative stress and better demonstrate the antioxidant effect of puerarin at the site of muscle atrophy. This will be a key focus in our future investigations. Additionally, the research is confined to the preclinical stage, and the safety, efficacy, and potential application of puerarin in the treatment of human sarcopenia still require validation through broader clinical trials. Therefore, future research should expand the scope of the study and evaluate its treatment effects and safety in clinical settings.

## 5 Conclusion

**FIGURE 9:**
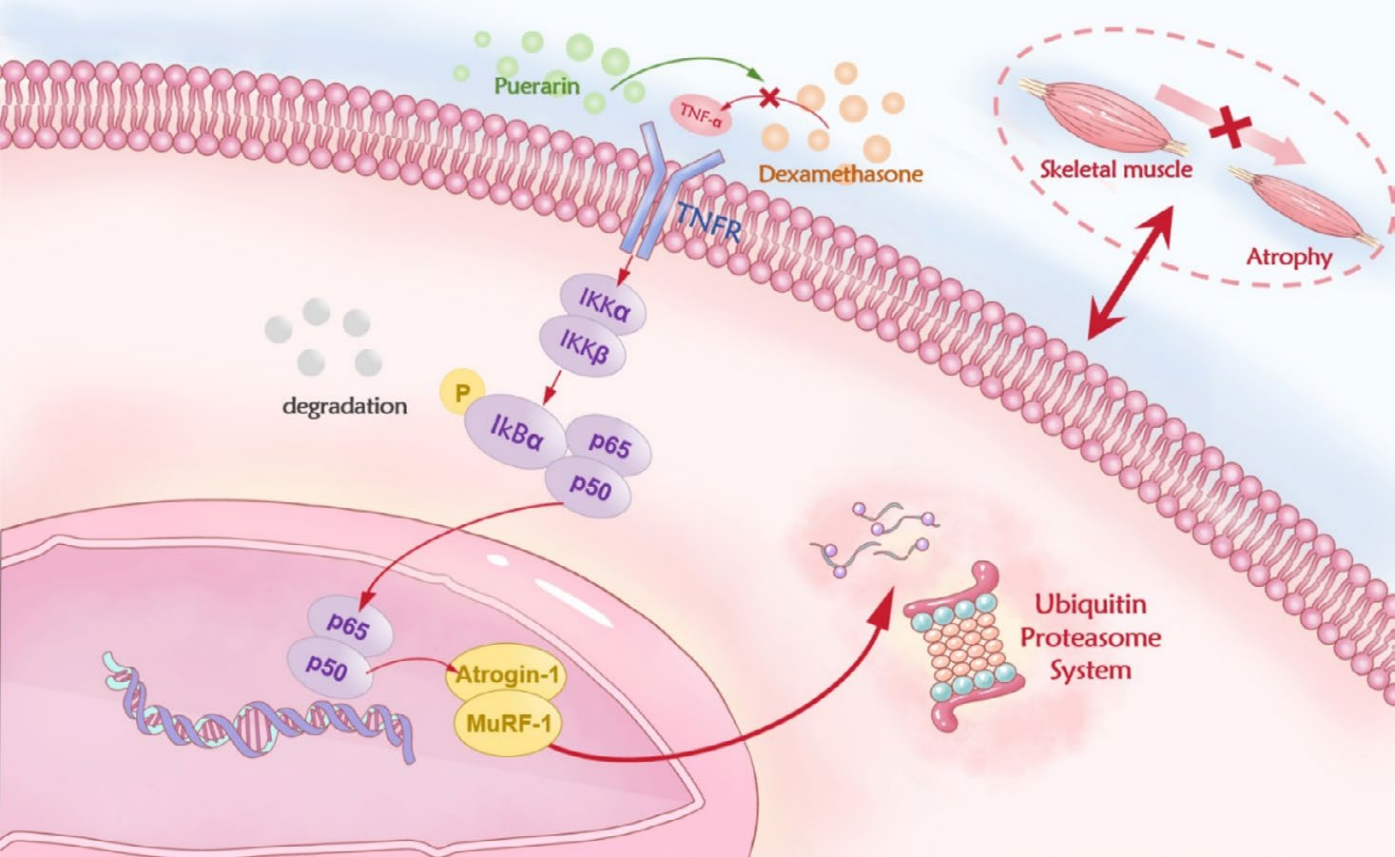
Schematic diagram of puerarin alleviating dexamethasone‐induced muscle atrophy via the TNF‐α/NF‐κB pathway.

In summary, this study demonstrated that puerarin effectively alleviates a range of physiological and behavioral alterations induced by dexamethasone, including weight loss, a decline in muscle mass, deterioration of muscle function, and damage to the ultrastructure of muscle fibers. A schematic diagram illustrating the proposed mechanism has been summarized in Figure 9. Notably, puerarin significantly enhances the muscle mass and function of affected mice, reduces the release of pro‐inflammatory factors while promoting the production of anti‐inflammatory factors, decreases oxidative stress, suppresses the expression of muscle apoptosis‐related proteins, and delays the progression of muscle atrophy by inhibiting the TNF‐α/NF‐κB signaling pathway.

## Author Contributions

**Shangjin Lin:** data curation (equal), formal analysis (equal), investigation (equal), methodology (equal), software (equal), validation (equal), writing – original draft (equal). **Ying Cheng:** data curation (equal), formal analysis (equal), methodology (equal), resources (equal), software (equal), validation (equal), writing – original draft (equal). **Xiuxiu Chen:** formal analysis (equal), investigation (equal), methodology (equal), resources (equal), software (equal), validation (equal), visualization (equal). **Fengjian Yang:** data curation (equal), investigation (equal), software (equal), validation (equal), visualization (equal). **Yongqian Fan:** conceptualization (equal), funding acquisition (equal), investigation (equal), project administration (equal), supervision (equal), validation (equal), visualization (equal), writing – review and editing (equal). **Shengwu Yang:** conceptualization (equal), investigation (equal), methodology (equal), project administration (equal), supervision (equal), validation (equal), visualization (equal), writing – review and editing (equal).

## Ethics Statement

All experimental procedures performed in this study strictly adhered to the guidelines and were approved by the Research Ethics Committee of the School of Life Sciences at Fudan University (Approval No. 2022110016S).

## Conflicts of Interest

The authors declare no conflicts of interest.

## Supporting information


**Figure S1.** Crystal violet staining showing the effects of different concentrations of puerarin on dexamethasone‐induced myotube atrophy in C2C12 cells.

**Table S1.** Details regarding the antibodies utilized in the study.
**Table S2.** Sequence information of qRT‐PCR primers.

## Data Availability

No new datasets were generated during the course of this research. All data supporting the findings of this study are contained within the manuscript itself.
