# Supplementary material for: Puerarin Delays the Progression of Muscle Atrophy in Mice With Dexamethasone‐Induced Sarcopenia Through Inhibiting the TNF‐α/NF‐κB Pathway
Source: Food Sci Nutr. 2025 Apr 18;13(4):e70166. doi: 10.1002/fsn3.70166 (PMC12006924; doi:10.1002/fsn3.70166)
Supplement: Supplementary file 1 — Figure S1. Crystal violet staining showing the effects of different concentrations of puerarin on dexamethasone‐induced myotube atrophy in C2C12 cells. [file FSN3-13-e70166-s001.docx]

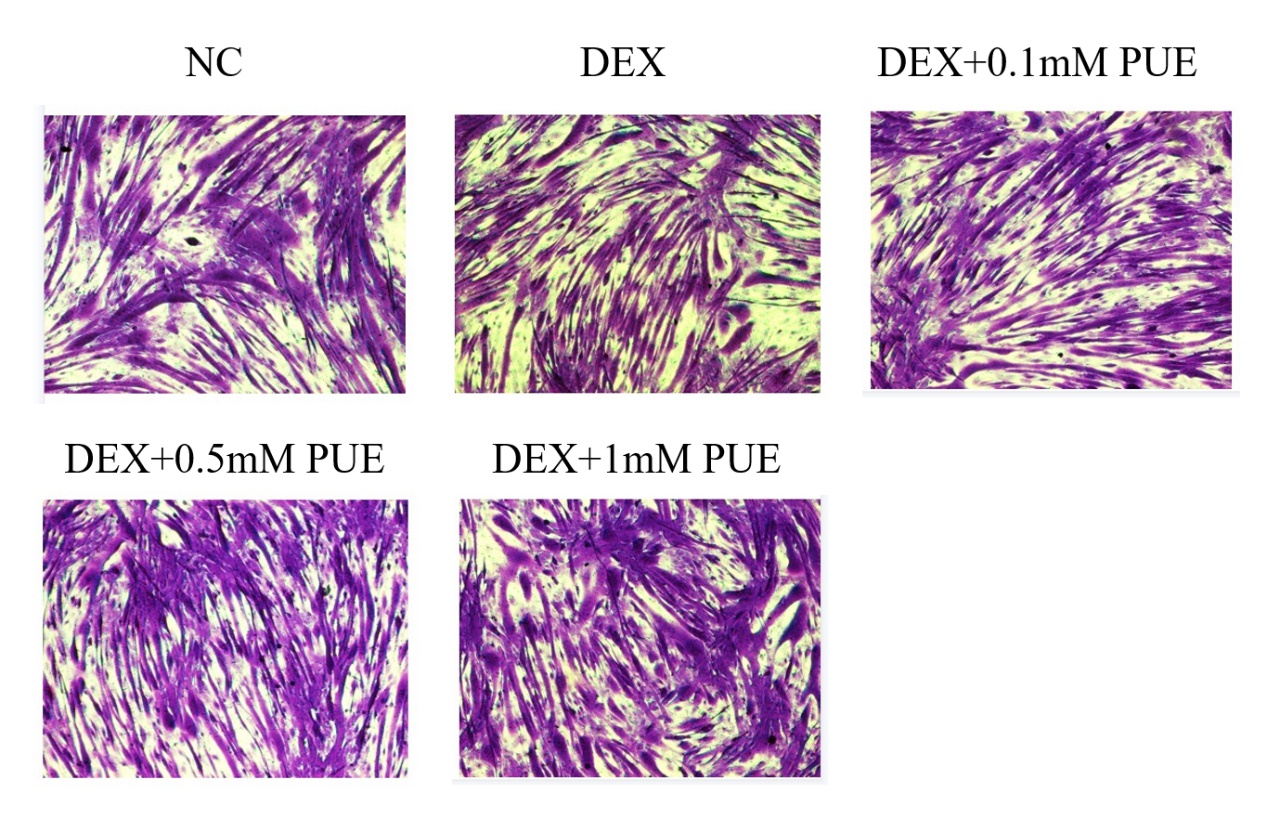
**Supplementary Fig**:

Fig. S1. Crystal violet staining showing the effects of different concentrations of puerarin on dexamethasone-induced myotube atrophy in C2C12 cells.
