# Supplementary material for: Puerarin Delays the Progression of Muscle Atrophy in Mice With Dexamethasone‐Induced Sarcopenia Through Inhibiting the TNF‐α/NF‐κB Pathway
Source: Food Sci Nutr. 2025 Apr 18;13(4):e70166. doi: 10.1002/fsn3.70166 (PMC12006924; doi:10.1002/fsn3.70166)
Supplement: Supplementary file 2 — Table S1. Details regarding the antibodies utilized in the study. Table S2. Sequence information of qRT‐PCR primers. [file FSN3-13-e70166-s002.docx]

Supplementary Table 1. Details regarding the antibodies utilized in the study.

| **Antibody Name** | **Company Name** | **Catalog** |
| --- | --- | --- |
| Anti-fast myosin skeletal heavy chain | Service Bio | GB112130 |
| Anti-slow skeletal myosin heavy chain | Service Bio | GB112131 |
| Anti-Atrogin-1 | Abcam | ab168372 |
| Anti-MuRF-1 | SANTA CRUZ | sc-398608 |
| Anti-HSP90 | CST | 4874S |
| Anti-β-Tublin | CST | 15115S |
| Anti-Bax | CST | 2772S |
| Anti-Bcl-2 | CST | 15071S |
| Anti-TNF-α | CST | 3707S |
| Anti-IKKα | Abclonal | A21354 |
| Anti-IKKβ | Abclonal | A22425 |
| Anti-phospho-IKKα/β | CST | 2697S |
| Anti-phospho-P65 | Abclonal | AP0123 |
| Anti-P65 | Abclonal | A21593 |
| Anti-phospho-IκBα | Abclonal | AP0614 |
| Anti-IκBα | Abclonal | A19714 |
| Anti-rabbit/mouse IgG, HRP-linked Antibody | CST | 7074S/7076S |
| Fluorescent secondary antibodies (donkey/goat) | Service Bio | GB21403/GB25303 |

Supplementary Table 2. Sequence information of qRT-PCR primers

| **Gene** | **Primer type** | **Primer sequence** |
| --- | --- | --- |
| **Myh1** | Forword | GCGAATCGAGGCTCAGAACAA |
|  | Reverse | GTAGTTCCGCCTTCGGTCTTG |
| **Myh2** | Forword | AAGTGACTGTGAAAACAGAAGCA |
|  | Reverse | GCAGCCATTTGTAAGGGTTGAC |
| **Myh4** | Forword | CTTTGCTTACGTCAGTCAAGGT |
|  | Reverse | AGCGCCTGTGAGCTTGTAAA |
| **Myh7** | Forword | ACTGTCAACACTAAGAGGGTCA |
|  | Reverse | TTGGATGATTTGATCTTCCAGGG |
| **Atrogin-1** | Forword | CAGCTTCGTGAGCGACCTC |
|  | Reverse | GGCAGTCGAGAAGTCCAGTC |
| **Murf-1** | Forword | GTGTGAGGTGCCTACTTGCTC |
|  | Reverse | GCTCAGTCTTCTGTCCTTGGA |
| **Gapdh** | Forword | CGTGCCGCCTGGAGAAAC |
|  | Reverse | TGGGAGTTGCTGTTGAAGTCG |
